# Supplementary material for: Global warming due to loss of large ice masses and Arctic summer sea ice
Source: Nat Commun. 2020 Oct 27;11:5177. doi: 10.1038/s41467-020-18934-3 (PMC7591863; doi:10.1038/s41467-020-18934-3)
Supplement: Supplementary file 1 — Supplementary Information [file 41467_2020_18934_MOESM1_ESM.pdf]

Supplementary Information

Global warming due to loss of large ice masses and Arctic summer sea ice

Wunderling et al.

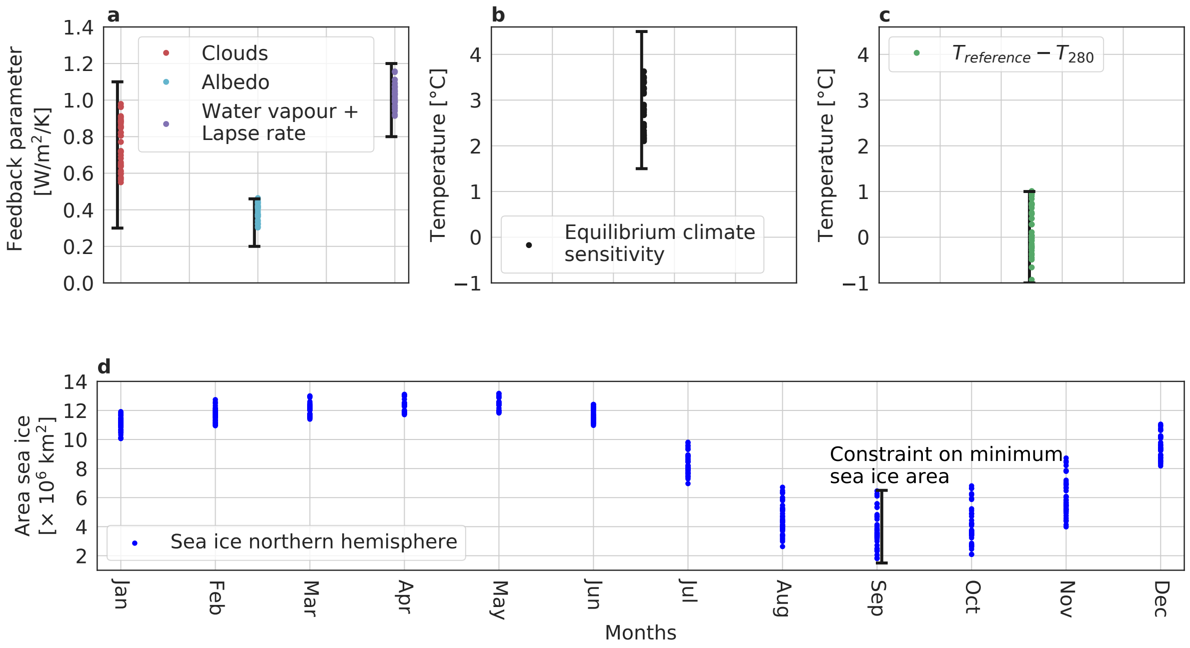

Supplementary Figure 1 | CLIMBER-2-GCM calibration. a, Calibration measurements for the cloud (red), the albedo (cyan) and the water vapour together with the lapse rate feedback (purple) constrained using the GCMs from Soden & Held(2006)[1]. The exact values that we constrained for are 0.8 – 1.2 W/m^2/K for the water vapour together with the lapse rate feedback, 0.3 – 1.1 W/m^2/K for the cloud feedback and 0.2 – 0.45 W/m^2/K for the albedo feedback. Note that not all climate feedbacks cover the whole range of possible limits shown as black errorbars[1,2]. **b**, An additional constraint due to the equilibrium climate sensitivity when CO_2 concentration is doubled (black) is applied. The equilibrium climate sensitivity is restricted to 1.5–4.5 °C. **c**, Another constraint (green) is applied for a reference run at a concentration of $p(CO_2) = 280$ ppm compared with a perturbed parameter run at the same CO_2 concentration. This results in: $|T_{\text{reference}} - T_{280}| \leqslant 1,0$°C. **d**, A final constraint is applied to the minimum sea ice area in the northern hemisphere which is supposed to be between $1.5-6.5\times10^6$ km^2. This constraint is applied to prevent too low sea ice areas at a CO_2 concentration of 280 ppm. For all the calibration runs, the parameters from supplementary Table 1 were varied.

Parameter	Varied range (Mean)	Source
Γ_0: Temp. lapse rate parameter	$4.7 - 5.2$ (5.0) $\cdot 10^{-3}$	Eq. 2[3]
Γ_1: Temp. lapse rate parameter	$3.6 - 4.4$ (4.0) $\cdot 10^{-5}$	Eq. 2[3]
Γ_2: Temp. lapse rate parameter	$0.7 - 1.3$ (1.0) $\cdot 10^{-3}$	Eq. 2[3]
OD_1: Cloud optical depth parameter	$9.5 - 10.5$ (10.0)	App. 7.3[4]
OD_2: Cloud optical depth parameter	$7.5 - 8.5$ (8.0)	App. 7.3[4]
$D(A_{CO21})$: Integral transmission of atm.	$0.3 - 0.65$ (0.5)	Eq. 3[3]
c_1: Cloudiness height parameter	$0.178 - 0.188$ (0.183)	Eq. 34[3]
α_{snow}: Diffusive new snow albedo	$0.85 - 1.0$ (0.95)	Fig. 2[5]

Supplementary Table 1 | Parameters for calibration of CLIMBER-2. Varied parameters in CLIMBER-2 in order to reconstruct GCM-uncertainty ranges of feedbacks[1]. Procedure of varying parameters similar to Deimling et al.(2006)[4].

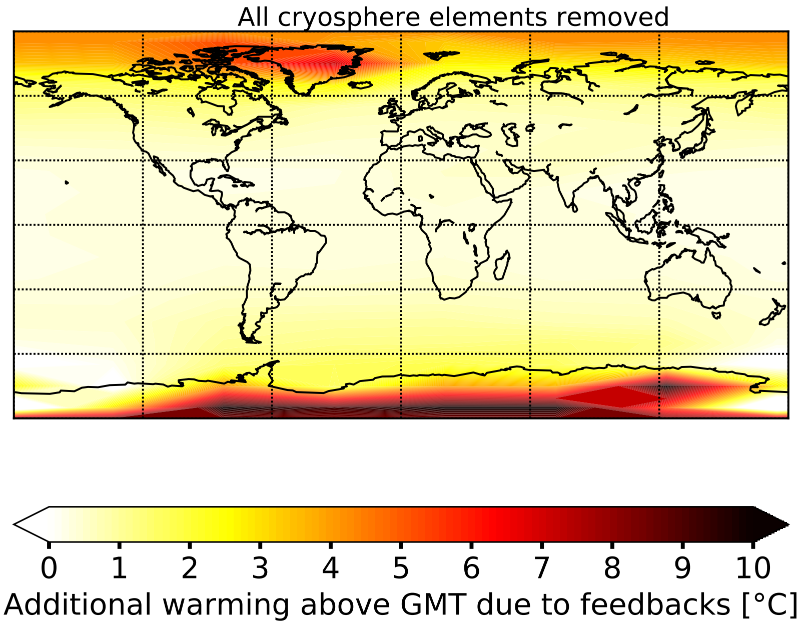

Supplementary Figure 2 | Warming including the Antarctic Ice Sheet. For a better comparability to literature[6,7], we ran a simulation, where we removed all the cryosphere elements: West Antarctica, Greenland, Arctic summer sea ice, mountain glaciers and East Antarctica. We find a strong warming in the proximity of the locations where ice is removed, which is in good agreement with literature, see also Fig. 4e in Lunt et al. (2012)[7]. However, both studies from the literature used an ice sheet reconstruction for the late Pliocene (PRISM), which includes the loss of most of Greenland and West Antarctica, but also of substantial parts of East Antarctica. With these prescribed ice sheets Lunt et al. (2012)[7] found a global warming of 0.7 °C, where we find 0.2 °C, but the discrepancy comes mainly from East Antarctica, which is still intact in our simulations in the main manuscript (see Fig. 1). With the additional removal of the East Antarctic Ice Sheet at a CO_2 concentration of 280 ppm, we find an additional warming of 0.82 °C.

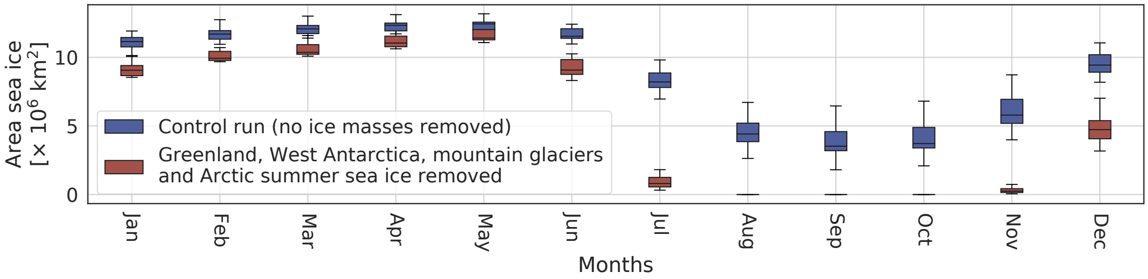

Supplementary Figure 3 | Sea ice area in the northern hemisphere. Loss of sea ice in the control (intact cryosphere components) and perturbed run for a CO_2 concentration of 280 ppm over the course of one year. In the perturbed run Greenland, West Antarctica, the mountain glaciers were removed and the albedo area of the Arctic summer sea ice was replaced by the ocean albedo during June, July and August. The main values indicate the median of the ensemble, the boxes show the interquartile range and the error bars depict the full ensemble spread.

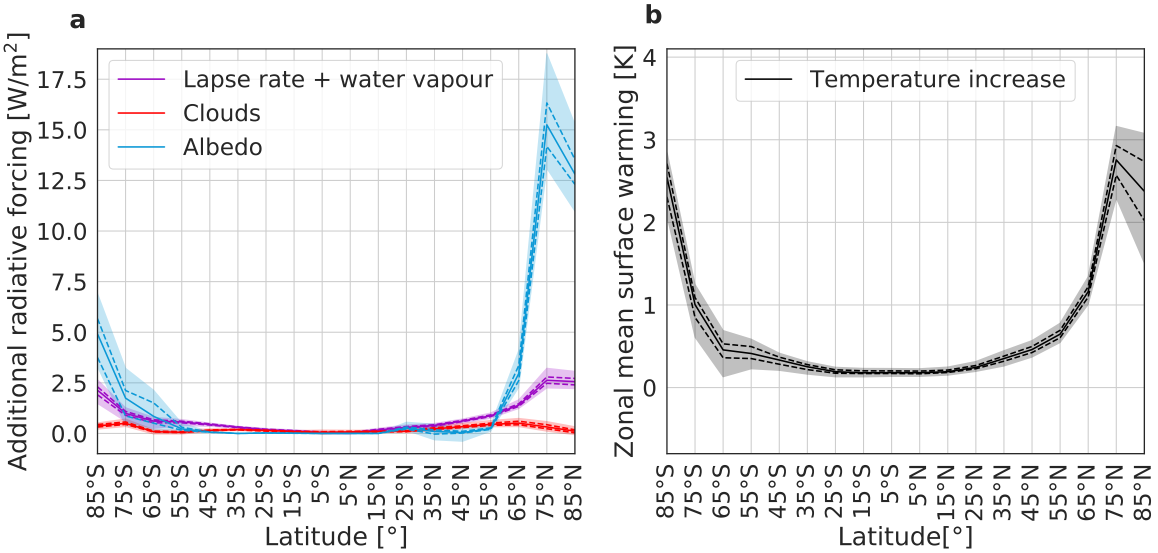

Supplementary Figure 4 | Zonal distribution of additional radiative perturbation and surface warming. a, Contributions from different causes of additional radiative perturbation, where we compare the perturbed run (without cryosphere elements) with the control run (with cryosphere elements) for all 39 runs at a CO_2 concentration of 400 ppm. The additional radiative perturbation is strongest in the polar regions. **b**, Zonal mean surface warming over latitude with a strong warming in polar regions. The errors are given as the full ensemble spread in shaded colours, the straight line denotes the median of the ensemble and the dashed lines show the interquartile range.

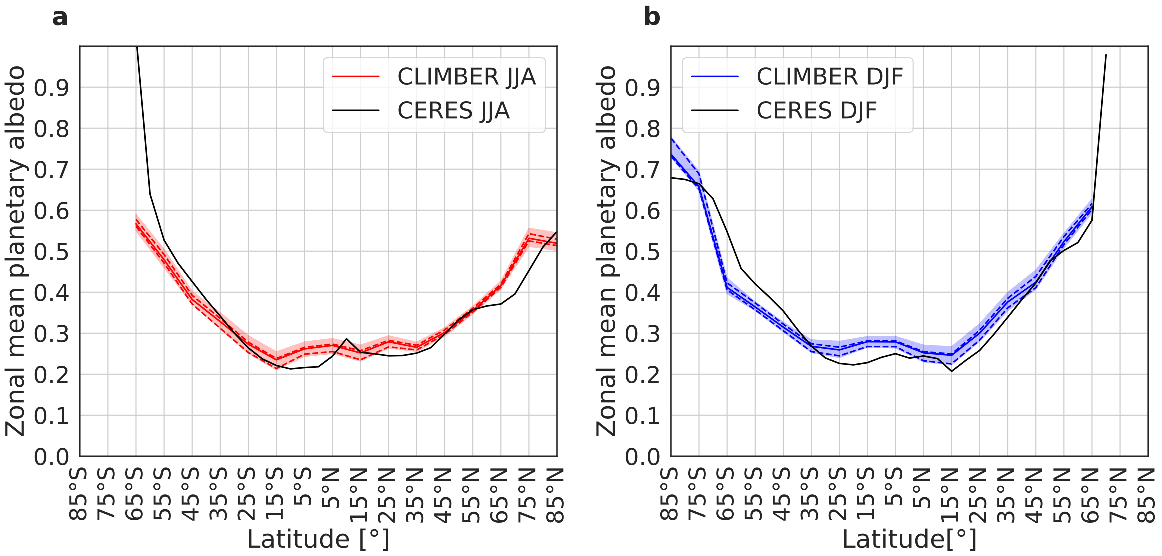

Supplementary Figure 5 | Comparison of zonal mean of planetary albedo between CERES and CLIMBER-2. Planetary albedo in CLIMBER-2 and CERES data[8] are in good agreement, **a,** for the months JJA and **b,** for the months DJF. The CLIMBER-2 data is shown as the full ensemble spread, where the straight line indicates the median of the ensemble, the dashed line the interquartile range and the shaded area the full ensemble spread. The data is cut at 65 °S during JJA since no planetary albedo values are available at this time due to the Antarctic night. The CERES data deviates at this edge from the CLIMBER data due to the observational sparse data close to the Antarctic night region. For the same reason, the data is cut due to the Arctic night in panel **b**.

Supplementary References

1. Soden, B. J. & Held, I. M. An assessment of climate feedbacks in coupled ocean–atmosphere models. *Journal of climate* **19**, 3354–3360 (2006).

2. Stocker, T. F. *et al.* Climate change 2013: The physical science basis. *Contribution of working group I to the fifth assessment report of the intergovernmental panel on climate change* **1535** (2013).

3. Petoukhov, V. *et al.* Climber-2: a climate system model of intermediate complexity. part i: model description and performance for present climate. *Climate dynamics* **16**, 1–17 (2000).

4. von Deimling, T. S., Held, H., Ganopolski, A. & Rahmstorf, S. Climate sensitivity estimated from ensemble simulations of glacial climate. *Climate Dynamics* **27**, 149–163 (2006).

5. Rösel, A., Kaleschke, L. & Birnbaum, G. Melt ponds on arctic sea ice determined from modis satellite data using an artificial neural network. *The cryosphere* **6**, 431–446 (2012).

6. Lord, N. S. *et al.* Emulation of long-term changes in global climate: application to the late pliocene and future. *Climate of the Past* **13**, 1539–1571 (2017).

7. Lunt, D. J. *et al.* On the causes of mid-pliocene warmth and polar amplification. *Earth and Planetary Science Letters* **321**, 128–138 (2012).

8. Wielicki, B. A. *et al.* Clouds and the earth's radiant energy system (ceres): An earth observing system experiment. *Bulletin of the American Meteorological Society* **77**, 853–868 (1996).
